# DYRK1a Inhibitor Mediated Rescue of *Drosophila* Models of Alzheimer’s Disease-Down Syndrome Phenotypes

**DOI:** 10.3389/fphar.2022.881385

**Published:** 2022-07-19

**Authors:** Bangfu Zhu, Tom Parsons, Wenche Stensen, John S. Mjøen Svendsen, Anders Fugelli, James J. L. Hodge

**Affiliations:** ^1^ School of Physiology, Pharmacology and Neuroscience, Faculty of Life Science, University of Bristol, Bristol, United Kingdom; ^2^ Department of Chemistry, The Arctic University of Norway, Tromsø, Norway; ^3^ Pharmasum Therapeutics AS, ShareLab, Forskningsparken i Oslo, Oslo, Norway

**Keywords:** *Drosophila*, sleep, memory, tau, amyloid-β, DYRK1A, minibrain, kinase inhibitor

## Abstract

Alzheimer’s disease (AD) is the most common neurodegenerative disease which is becoming increasingly prevalent due to ageing populations resulting in huge social, economic, and health costs to the community. Despite the pathological processing of genes such as *Amyloid Precursor Protein* (*APP*) into Amyloid-β and *Microtubule Associated Protein Tau* (*MAPT*) gene, into hyperphosphorylated Tau tangles being known for decades, there remains no treatments to halt disease progression. One population with increased risk of AD are people with Down syndrome (DS), who have a 90% lifetime incidence of AD, due to trisomy of human chromosome 21 (HSA21) resulting in three copies of *APP* and other AD-associated genes, such as *DYRK1A* (Dual specificity tyrosine-phosphorylation-regulated kinase 1A) overexpression. This suggests that blocking DYRK1A might have therapeutic potential. However, it is still not clear to what extent DYRK1A overexpression by itself leads to AD-like phenotypes and how these compare to Tau and Amyloid-β mediated pathology. Likewise, it is still not known how effective a DYRK1A antagonist may be at preventing or improving any Tau, Amyloid-β and DYRK1a mediated phenotype. To address these outstanding questions, we characterised *Drosophila* models with targeted overexpression of human *Tau*, human *Amyloid-β* or the fly orthologue of *DYRK1A*, called *minibrain* (*mnb*). We found targeted overexpression of these AD-associated genes caused degeneration of photoreceptor neurons, shortened lifespan, as well as causing loss of locomotor performance, sleep, and memory. Treatment with the experimental DYRK1A inhibitor PST-001 decreased pathological phosphorylation of human Tau [at serine (S) 262]. PST-001 reduced degeneration caused by human Tau, Amyloid-β or mnb lengthening lifespan as well as improving locomotion, sleep and memory loss caused by expression of these AD and DS genes. This demonstrated PST-001 effectiveness as a potential new therapeutic targeting AD and DS pathology.

## Introduction

AD is the most common form of dementia with ∼50 million suffers worldwide which is set to double every 20 years with ageing populations. In the US alone the societal and economic cost of dementia is $818 billion (Alzheimer’s Association, 2016) and yet there remain no cure for AD, and only limited symptomatic treatments that includes anti-cholinesterases, a NMDA glutamate receptor antagonist and an Aβ clearing antibody, however these do not halt or reverse neurodegeneration. Loss of cholinergic neurons in the cortex and hippocampus is particularly pronounced leading to the hallmark behavioural changes such as personality changes and loss of sleep and memory which precede a shortening of life (Querfurth and LaFerl a, 2010; Selkoe and Hardy, 2016; Mukhopadhyay and Banerjee, 2021). Post-mortem examination of AD brains reveals neurodegeneration is highly correlated with accumulation of extracellular Amyloid-β (Aβ) plaques that are produced by the amyloidogenic cleavage of APP by secretases generating aggregating Aβ peptides such as the 42 amino acid (aa) peptide (Aβ42) (Selkoe and Hardy, 2016). The cognitive deficits of AD are most strongly correlated with the accumulation of intracellular tangles of hyperphosphorylated Tau. These are encoded by the microtubule associated protein Tau (MAPT) gene that can be alternatively spliced into six main isoforms of Tau which vary in numbers of both N-terminal domains (e.g., 0, 1, or 2N) and C-terminal aggregating tubulin-binding repeats (e.g., 3R or 4R). 4R isoforms are thought to display stronger tubulin binding and aggregation than 3R isoforms and are upregulated in the AD brain (Arendt et al., 2016). Tau aggregation is increased by hyperphosphorylation by several different kinases including glycogen synthase kinase-3β, cyclin-dependent kinase 5, JNK, microtubule-associated regulatory kinase, CaMKII and DYRK1A (Hanger et al., 1992; Ferrer et al., 2005; Plattner et al., 2006; Wang et al., 2007; Hooper et al., 2008; Dolan and Johnson, 2010; Ghosh and Giese, 2015; Toral-Rios et al., 2020).

Only 5% of the causes of AD are thought to be genetic, making it difficult to predict who will develop the disease or not, with diagnosis only usually possible after extensive and irreversible neurodegeneration has occurred frustrating attempts at early intervention and development of effective treatments (Querfurth and LaFerl a, 2010; Selkoe, 2012; Selkoe and Hardy, 2016; Congdon and Sigurdsson, 2018; Soeda and Takas hima, 2020). However, one large cohort of people almost certain to get AD are those with DS or trisomy 21 which is caused by three copies of HSA21 as opposed to the normal two, with AD causal gene, APP located on HSA21, hence three copies of APP leading to increased likelihood of AD pathology (Selkoe and Hardy, 2016; Herault et al., 2017). DS can be diagnosed *in utero,* is common (1:450-1:2,200 live births) and is characterised by a range of developmental abnormalities associated with loss of motor skills, learning ability and sleep (Lott and Dierssen, 2010; Malak et al., 2015). People with DS have an 90% chance of developing Alzheimer’s disease (AD)-like symptoms (AD-DS) that includes progressive dementia from about 40 years old preceded by Aβ plaques and neurofibrillary tangles (NFTs) of Tau accumulation resulting in neurodegeneration from about 10 to 20 years old (Zigman, 2013; Wiseman et al., 2015). This pathology leads to the AD symptoms of progressive motor, cognitive and health decline (Anderson-Mooney et al., 2016) and shortening of life by ∼30 years (O'Leary et al., 2018).

However, another HSA21 gene, *DYRK1A* can also be considered a candidate causal gene for the pathological changes that occur in the DS and AD brain resulting in the associated cognitive and motor deficits (Ferrer et al., 2005; Wiseman et al., 2015; García-Cerro et al., 2017; Herault et al., 2017; Arbones et al., 2019). DYRK1A is highly expressed throughout the brain with increased levels in DS (Duchon and Herault, 2016; Kay et al., 2016). Therefore, the large alterations in DYRK1A gene-dosage are thought to have particularly negative consequences in DS, that may overshadow the more subtle effect it may have later in life that increase the risk of developing AD. DYRK1A has been genetically linked to late-onset AD (LOAD) within the Japanese population (Kimura et al., 2007). Furthermore people with DS having more than 100x risk of developing AD compared to rest of population, again largely thought to be due overexpression of APP and other chromosome 21 genes like DYRK1A throughout development and adulthood, leading to AD pathology including amyloid plaques and Tau tangles in young adults (Zigman, 2013; Wiseman et al., 2015; Selkoe and Hardy, 2016). However, in induced pluripotent stem cells from people with DS, the role of APP trisomy was demonstrated to be disconnected from tau pathology and neuronal cell death (Ovchinnikov et al., 2018). This suggests that DYRK1A and tau pathology is a larger contributor to AD-DS, than APP (Wiseman et al., 2018).

Regardless of the differing potential roles of APP, tau and DYRK1A in DS, AD-DS, and AD, there remains, no effective treatments for any of these diseases. This reflects the lack of knowledge of underlying mechanisms for these diseases and a deficit of new models, which is slowing progress and especially in development of new targets and treatments whose efficacy might translate to patients. Most of our understanding of the mechanistic changes that cause AD pathology comes from experiments performed in rodent familial AD models involving knock-ins or misexpression of different APP or Tau transgenes however these are not thought to recapitulate the sporadic disease which make up ∼95% of causes of AD. These factors have contributed to the large drug attrition of new drugs that although effective in these rodent models have not translated to any new AD treatments from clinical trials emphasising the need for new molecular models of sporadic AD (McGowan et al., 2006; Crews and Masliah, 2010; Van Dam and De Deyn, 2011; Gama Sosa et al., 2012; Guo et al., 2012; De Jager et al., 2014). Mouse models overexpressing *Dyrk1a* disrupt brain and eye development causing recapitulating cognitive and motor deficits seen in DS (Altafaj et al., 2001; Ahn et al., 2006; Guedj et al., 2012; Laguna et al., 2013; García-Cerro et al., 2014; Duchon and Herault, 2016). DYRK1A is also known to phosphorylate Tau at multiple sites including serine 262 that results in aggregation of pathological Tau neurofibrillary tangles associated with sporadic AD (Woods et al., 2001; Liu et al., 2008; Shi et al., 2008; Azorsa et al., 2010; Frost et al., 2011; Wegiel et al., 2011; Tenreiro et al., 2014). Increased DYRK1A also phosphorylates APP and promotes pathological processing of APP into neurotoxic Aβ peptides (Ferrer et al., 2005; García-Cerro et al., 2017; Arbones et al., 2019), overexpressing DYRK1A mice displaying AD pathology including neurodegeneration, disrupted synaptic plasticity and memory (Ferrer et al., 2005; Ahn et al., 2006; Liu et al., 2008; García-Cerro et al., 2017).

In *Drosophila*, neuronal overexpression of different human APP products [including human tandem oligomerising secreted Aβ42 (Chen et al., 2014a)] and mutants cause degeneration of the photoreceptor neurons of the fly eye, shortened lifespan, change in neuronal excitability as well as movement, circadian, sleep, and learning deficits (Iijima et al., 2004; Chiang et al., 2010; Spere tta et al., 2012; Chen et al., 2014a; Blake et al., 2015; Ping et al., 2015; Tabuchi et al., 2015; Higham et al., 2019a). Likewise, neuronal overexpression of AD-associated human Tau isoforms also result in degeneration of the photoreceptor neurons, central brain neurodegeneration, shortened lifespan, movement, changes in neuronal excitability, circadian rhythm, sleep, and learning defects (Wittmann et al., 2001; Folwell et al., 2010; Iijima-Ando and Iijima, 2010; Kosmidis et al., 2010; Beharry et al., 2013; Papanikolopoulou and Skoulakis, 2015; Sealey et al., 2017; Higham et al., 2019a; Higham et al., 2019b; Buhl et al., 2019; Lowe et al., 2019).

The *Drosophila* ortholog of *DYRK1A* is *mnb*, and produces five *mnb* isoforms, *E-I*, which all contain a highly conserved kinase domain (Hong et al., 2012; Gramates et al., 2017) and have conserved function in neuronal morphology, growth, brain development and cognition (Tejedor et al., 1995; Guimerá et al., 1996; Hämmerle et al., 2003; Degoutin et al., 2013; Ori-McKenney et al., 2016; Shaikh et al., 2016). Mnb is presynaptically localised at developing synapses and reducing its expression changed presynaptic structure and impaired recycling of transmitter vesicles with *mnb*-*F* overexpression ameliorating the effects of reduced *mnb* expression (Chen et al., 2014b). Gal4-mediated neuronal overexpression of *mnb-H* resulted in a significant increase in *mnb-H* expression, which is the isoform with the longest coding region (Hong et al., 2012; Gramates et al., 2017; Zerbino et al., 2018). Gal4-mediated neuronal overexpression of *mnb-H* was shown to cause motor impairment during development and ageing, shortened lifespan and resulted in age-related neurodegeneration with synaptic analysis showing increased number of glutamatergic boutons, enhanced spontaneous vesicular transmitter release, and slowed recovery from short-term depression (Lowe et al., 2019).

DYRK1A kinase antagonists have shown therapeutic potential for treatment for DS and AD in animal models (Smith et al., 2012; Duchon and Herault, 2016; Arbones et al., 2019). SM07883 DYRK1A inhibitor reduced pathological phosphorylation of Tau (including S212) in mice overexpressing MAPT P301L (associated with Frontal Temporal Lobe Dementia) reducing aggregation of Tau, neurodegeneration and improving behavioural deficits (Melchior et al., 2019). Leucettine DYRK1A inhibitor treatment of DYRK1A overexpressing mice rescued their cognitive deficits *via* correcting brain connectivity and expression of synaptic proteins (Nguyen et al., 2018). In 3xTg-AD mice that overexpress mutant Swedish APP (AD causal mutation), MAPT P301L and Presenilin M146V (AD causal mutation), a DYRK1A benzimidazole-like inhibitor reversed cognitive deficits *via* decreasing Aβ42 aggregation and decreasing phosphorylation of insoluble Tau (Branca et al., 2017).

Due to human DYRK1A and fly mnb sharing 82% amino acid identity which is even greater the kinase domains, many of the phenotypes and pharmacological sensitivity is conserved across species. For instance, conserved loss of function mutations reduced brain size in humans and flies, hence the name minibrain (Hämmerle et al., 2003; Lee et al., 2020a). With the DYRK1A-E396term being demonstrated to be loss of function mutation as it decreases the canonical phosphorylation of human Tau by DYRK1A. When the conserved mutation (mnb-D401term) was expressed in flies it removed the additive neurotoxic effect of co-expression of mnb and human Tau on eye degeneration (Lee et al., 2020a). Furthermore, a DYRK1A ATP-binding site competitive inhibitor, called CX-4945 reversed DYRK1A overexpression mediated increases in phosphorylation of Tau (S212), APP and PS1 *in vitro* and decreased degeneration of fly eyes overexpressing human Tau and decreased lethality caused by pan-neuronal developmental overexpression of mnb-H. The drug also suppressed pathological increases in Tau phosphorylation of mice overexpressing DYRK1A (Kim et al., 2016). This supports the potential of DYRK1A inhibition to treat DS and AD pathology. A recent highly potent and specific ATP-binding site small molecule DYRK1A inhibitor has been reported that crosses the blood brain barrier with 1 and 100 μM PST-001 significantly reducing DYRK1A activity reversing cognitive deficits of mouse DS models (Stensen et al., 2021a). Here we compare the effects of neuronal overexpression of fly *mnb-H,* human Tau (0N4R) and human tandem oligomerizing secreted Aβ42 on degeneration of adult photoreceptor neurons, longevity, motor performance, sleep, and memory and test the ability of a PST-001 DYRK1A inhibitor to suppress these phenotypes.

## Material and Methods

### Fly Stocks and Husbandry

Flies were raised at 25°C on 12 h light: 12 h dark (LD) cycles and on a standard corn yeast cornmeal diet (0.7% agar, 1.0% soya flour, 8.0% polenta/maize, 1.8% yeast, 8.0% malt extract, 4.0% molasses, 0.8% propionic acid, and 2.3% nipagen). *CSw* ^*-*^ wild type control flies were gifts from Dr. Scott Waddell (University of Oxford, United Kingdom) and were crossed with flies bearing *GAL4* transgenes with the heterozygous (*GAL4/+*) offspring being used as the control genotype. *Tim(27)-GAL4/CyO* flies (Buhl et al., 2016) were a gift from Dr. Ralf Stanewsky (University of Münster, Germany). The following strains were obtained from Bloomington *Drosophila* Stock Center (BDSC; stock number provided in brackets): *OK107-GAL4* (854), *GMR-GAL4/CyO* (9,146), *UAS-human MAPT (TAU 0N4R) wild-type* (gift from Dr. Linda Partridge, University College London) (Wittmann et al., 2001; Kerr et al., 2011), *UAS-human secreted tandem Aβ42-22 amino acid linker-Aβ42* (gift from Dr. Damian Crowther, University of Cambridge) (Spere tta et al., 2012; Chen et al., 2014a) and *UAS-mnb* flies [*minibrain-H*, CG42273 (Hong et al., 2012)] were kindly provided by Dr. Kweon Yu (Korea Research Institute of Bioscience and Biotechnology).

### Pharmacology

PST-001 DYRK1A inhibitor was manufactured by Pharmasum Therapeutics, 100 mg PST-001 (Molecular weight = 299.35 g/mol) was dissolved in 1 ml DMSO and then mixed into 1 L of cooling (∼40°C) liquid fly food yielding a final concentration of 334 μM. This drug concentration was based on the PST-001 concentration that was effective at suppressing associative memory deficits of Ts65 Dn Down syndrome model mice (Stensen et al., 2021a). Flies laid onto the food that contained drug compared to food that contained vehicle with the offspring being exposed to the drug throughout their development and adulthood including during testing.

### Western Blotting

Heads of flies panneuronally (*elav-Gal4*) overexpressing human Tau (0N4R) or control (*elav/+*) were dissected and put into RIPA Lysis Buffer (Thermo Scientific) containing 1/100 Protease/Phosphatase Inhibitor Cocktail (Cell Signalling). The heads were homogenized and spun down at 12,000 rpm for 15 min at 4°C. The supernatants were collected, and protein concentrations were determined using a NanoDrop spectrophotometer (Thermo Fisher Scientific).

Protein samples were then prepared by adding 1/4 of 4X Bolt™ LDS Sample Buffer (Thermo Scientific) and 1/10 of 10X Bolt™ Sample Reducing Agent (Invitrogen) and heated at 95°C for 3 min. Samples of 100 µg total protein were separated by sodium dodecyl sulphate–polyacrylamide gel electrophoresis using precast 4%–12% Bolt Mini gels and then transferred to PVDF membrane (Thermom Scientific). The membrane was blocked overnight at 4°C in blocking solution [Tris-buffered saline (TBS): 20 mmol/L Tris (pH 7.4), 150 mM NaCl, with 0.1% Tween 20 [TBS with Tween (TBST)] and 5% (wt/vol) bovine serum albumin (BSA)] and incubated at 4°C overnight with a primary antibody in TBST containing 1% BSA. The primary antibodies used were mouse anti-Tau (T9450, Sigma), mouse anti–β-actin (A2228, Sigma-Aldrich, 1:1,000), anti-Tau phospho S262, S356, S396, and T231 (Abcam) (1:500).

After three washes with TBST, the blots were incubated for 1 h at room temperature with horseradish peroxidase–conjugated mouse or rabbit IgG secondary antibody (1:2,000, Cell Signalling) and then washed three times with TBST. Detection was performed using Western ECL Substrate (GE) according to the manufacturer’s instructions and developed on X-ray films first and then scanned. The relative protein expression levels were quantified by densitometry using ImageJ Gel Analysis software. Western blots from at least three independent biological replicate experiments for each fly strain were used for quantification.

### Eye Degeneration Assay

Overexpression of transgenes was driven in the eye throughout development and adulthood using the *Glass multimer reporter* (*GMR-GAL4*) promoter to test for neurotoxicity. 2–5 day old adult flies were CO_2_ anesthetised before immersion in ethanol to euthanise the fly to prevent movement during image capture (Folwell et al., 2010). The eyes were imaged with a Zeiss AxioCam MRm camera attached to a stereomicroscope (Zeiss SteREO Discovery. V8, up to 8x magnification) and an image capture to show if the genotype displayed the qualitative phenotype of “rough eyes,” Surface area was quantified using Zeiss Zen software and Two-way ANOVA with multiple comparisons tests were used to analyse data.

### Survival Assay

Approximately 2 days after eclosion five sets of ten mated (they were housed with males for 24 h) females were transferred to a vial containing standard food with or without drug and maintained at 25°C. Deaths were scored every 2 days and the remaining flies transferred to a fresh food with or without drug vial (Kerr et al., 2011). Data was presented as Mantel-Cox survival curves with statistical analysis performed using log-rank tests to compare survival between genotypes.

### Climbing Assay

Five groups of ten 2–5 days old flies per genotypes were collected and given 1 h to acclimatise to a standard empty food vial at 25°C. Exploiting the negative geotaxis reflex of *Drosophila*, flies were gently tapped to the bottom of the 7.5 cm plastic vial and the number of flies that crossed a line drawn 2 cm from the top of the tube in 10 s was counted, and then expressed as a % which was referred to as the climbing performance (Iijima et al., 2004; Sun et al., 2018). Two-way ANOVA with Dunnett’s multiple comparisons was used to analyse data.

### *Drosophila* Activity Monitoring for Sleep

Sleep monitoring experiments were conducted as previously described (Buhl et al., 2019; Curran et al., 2019; Tasman et al., 2020). Briefly, flies (male, 2–5 days old) were transferred into single tubes and placed individually in the *Drosophila* Activity Monitoring (DAM) system (DAM2, TriKinetics Inc., United States). Sleep was measured from activity data from 5 days of 12 h LD, summed into 1 and 30 min bins. Sleep was defined as bouts of inactivity lasting more than 5 min as per convention (Hendricks et al., 2000; Shaw et al., 2002; Parisky et al., 2008). The mean total sleep, mean sleep in the day and night were calculated for each individual using the Sleep and Circadian Analysis MATLAB Program (SCAMP) in MATLAB (Donelson et al., 2012).

### Aversive Olfactory Conditioning

Olfactory memory experiments were performed as previously described (Malik et al., 2013; Hidalgo et al., 2021a; Hidalgo et al., 2021b). Groups of 20–40 (mixed sex) flies were collected about 2–5 days prior to testing and kept under LD 25°C and 70% relative humidity conditions to acclimatise to the environment they would be tested in. Behavioural testing was performed under dim red light, so the flies could concentrate on odour cues. Sensorimotor controls were performed to test the olfactory acuity and shock reactivity of flies on and off drug. For shock reactivity, flies were given the choice between two shock tubes that formed the arms of the T-maze, one of which delivered the shock as described above, the number of flies avoiding shock over the total number of flies in the assay were used to calculate % shock avoidance. In a similar manner the % avoidance of concentration of odour used above (octanol or MCH) versus air was calculated.

To test associative memory, flies were transferred into a training tube lined with an electrifiable grid, and after a 90 s period to acclimatise to the stream of fresh air, flies were then exposed to an odorant (conditioned stimulus, CS^+^) paired with twelve 70 V DC electric shocks (unconditioned stimulus, US) for 1 min. The flies were then exposed to a second odorant (CS^−^) without electric shock. The odorants used were either 3-octanol (OCT, Sigma) or 4-methylcyclohexanol (MCH, Sigma) which were diluted into 10 ml mineral oil and adjusted to a concentration that the flies found equally aversive. A 45 s period of fresh air exposure separated CS^+^ and the CS^−^ to clear any residual odour. Memory was evaluated at 1 h post-conditioning to test intermediate-term memory (ITM). A performance index (PI) was calculated using the following equation:

$$PI=\;\frac{\left(N_{CS-}-N_{CS+}\right)}{\left(N_{CS-}+N_{CS+}\right)}$$

where N_CS−_ and N_CS+_ is the number of flies choosing CS^−^ and CS^+^, respectively. The CS^+^ odour was reversed in alternate groups of flies to minimise any possible trial to trial innate bias toward one odorant. The average of the performance between these two consecutive trials was considered as a *n* = 1 (i.e., 40−80 flies).

### Statistical Analysis

Data were analysed using GraphPad Prism (version 8.00 for Windows, GraphPad Software, La Jolla California United States). Normality was assessed in all datasets using Shapiro-Wilk’s test, prior to choosing the appropriate parametric or non-parametric statistical test to be used. The description of the tests used and the number of experiments/animals (n) for each dataset are indicated in the corresponding figures. Data is presented as Mean ± Standard error of the mean (SEM). Statistical levels are denoted as following non-significant (ns) *p* > 0.05, *p < 0.05, **p < 0.01, ***p < 0.001, and ****p < 0.0001.

## Results

### PST-001 Inhibits Pathological Phosphorylation of Human Tau at S262

It is known that increased DYRK1A kinase activity is involved in neurodegeneration, including *via* Tau hyperphosphorylation and pathological changes in amyloid-β, and pharmacological inhibition of DYRK1A is able to suppress this pathology (Ferrer et al., 2005; Liu et al., 2008; Wegiel et al., 2008; Smith et al., 2012; Coutadeur et al., 2015; Kim et al., 2016; Branca et al., 2017; Nguyen et al., 2018; Melchior et al., 2019; Lee et al., 2020b). Hence, the effectiveness of the DYRK1A protein kinase inhibitor PST-001 (Stensen et al., 2021a) was explored. The molecule is designed on the 5-methoxybenzothiazole scaffold known to show a preferential binding to the DYRK-family of protein kinases (Rothweiler et al., 2016), but PST-001 is extended with an acetamidopyridine moiety to enhance its binding efficacy (Stensen et al., 2021b). Kinase profiling of PST-001 verified that the compound is very selective indeed, delivering a GINI-index of 0.936, with the other members of the DYRK-family as well as CLK2 as the most affected off-targets. Furthermore, PST-001 is void of activity against the protein kinase GSK3β, that is involved in Tau phosphorylation and NFT formation (Toral-Rios et al., 2020) as well as contributing to the effects of AD through the Wnt pathway (Hooper et al., 2008), enabling the separation of DYRK1A mediated effects over GSK3β effects in *in vivo* models. The PST-001 molecule was designed to be orally active and to penetrate the blood-brain barrier, hence, to be an effective tool compound for investigating the effects of DYRK1A inhibition *in vivo.* A concentration of 100 mg PST-001/kg food was verified to give a therapeutic level of the drug in the brain of Ts65 Dn DS model mice suppressing their associative memory deficits (Stensen et al., 2021a). Mouse DYRK1a and mnb are highly conserved in evolution with 82% aa identity and with the ATP pocket and binding site of PST-001 being particularly highly conserved (Shindoh et al., 1996; Aranda et al., 2011) making it is highly likely that an active site DYRK1A inhibitor will also inhibit mnb.

**FIGURE 1:**
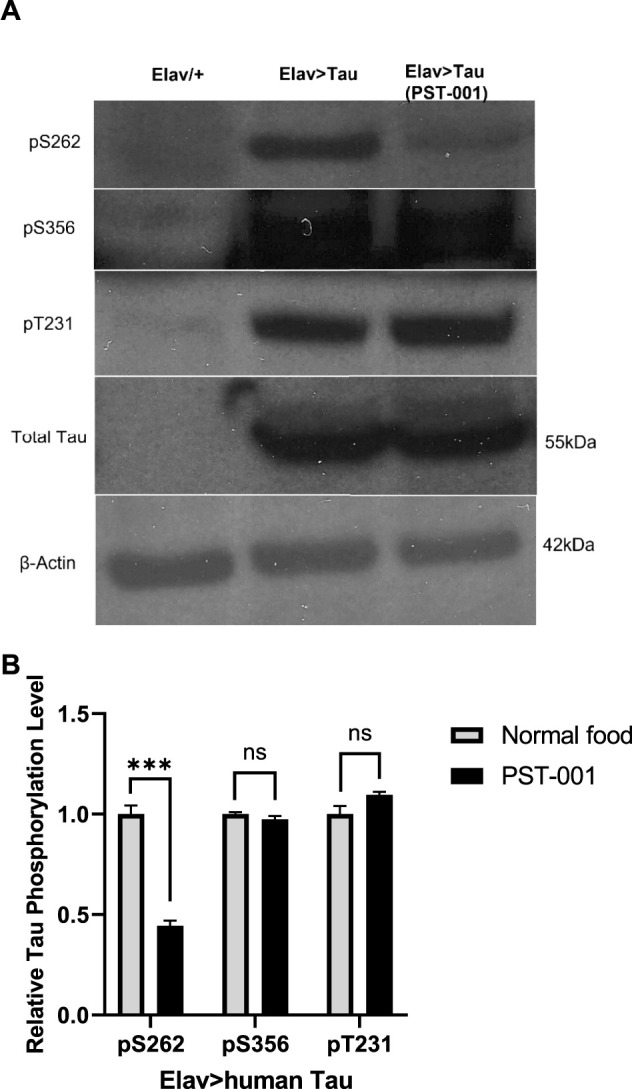
DYRK1A inhibitor PST-001 reduces phosphorylated human Tau expressed in *Drosophila* neurons. **(A)** Western blots show effects of PST-001 treatment on phosphorylation of human Tau 0N4R overexpressed pan-neuronally by *elav-Gal4* driver. 1^st^ lane is *Elav/+* genetic control, 2^nd^ lane *Elav>human Tau* on normal food and 3^rd^ lane *Elav>human Tau* with PST-001 treatment. Three antibodies against phosphorylated human Tau (pS262, pS356, and pT231) and one against total human Tau (∼55 kDa) were tested. β-actin (∼42 kDa) was used as a protein loading control. **(B)** Quantification of intensity of bands for human Tau phosphorylated at site S262, S356, and T231, in the presence and absence of PST-001.

Therefore, we fed 334 μM PST-001 to control (*elav/+*) and flies panneuronally overexpressing human Tau 0N4R (*elav>Tau*) throughout development and adulthood. Whole brain lysates were prepared for Western blotting with antibodies specific to human Tau and phospho-specific antibodies to Tau S262, S356, S396, and T231, phosphorylation events that lead to AD pathology (Azorsa et al., 2010; Frost et al., 2011; Tenreiro et al., 2014). *Elav>Tau* flies showed robust overexpression of human Tau detected with the human Tau antibody (Figure 1). There was little reactivity evident to endogenous fly Tau in the control lane (*elav/+*), all samples displayed equal loading of total protein as confirmed by the β-actin protein loading control. Treatment with the DYRK1A specific kinase inhibitor PST-001, did change the total amount of human Tau expressed, but caused a significant reduction (*p* < 0.001, *t*-test) of over 50% in the level of phosphorylated Tau at S262. A site that has been previously shown to be phosphorylated by DYRK1A leading to pathological aggregation of Tau in AD (Azorsa et al., 2010; Frost et al., 2011; Tenreiro et al., 2014). No significant changes of Tau phosphorylation were detected at the other tested sites (S356 and T231). This is consistent with PST-001 also being an inhibitor of the fly ortholog of DYRK1A, mnb.

### The PST-001 DYRK1A Inhibitor Decreases Photoreceptor Neuron Degeneration of Alzheimer Disease-Down’s Syndrome Model Flies

**FIGURE 2:**
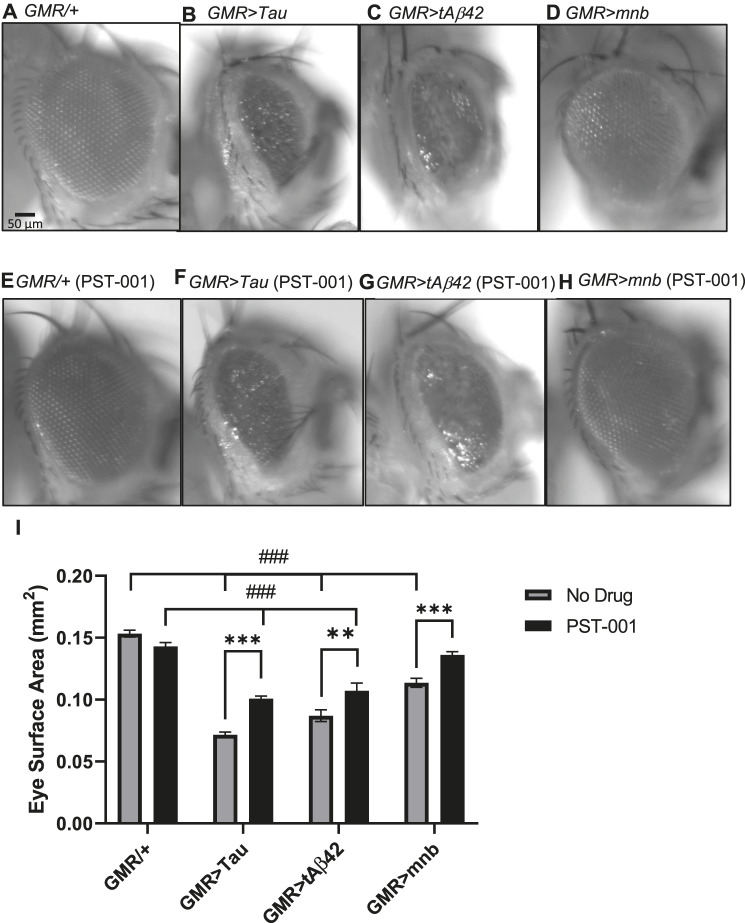
Degeneration of the eye caused by human Tau, amyloid-β42 or mnb overexpression can be reduced by the PST-001 DYRK1A inhibitor. **(A)** (*Left*) Representative images of the eyes of control adult flies (*GMR-GAL4/+*; scale bar: 50 μm) and flies overexpressing in the photoreceptor neurons of the eye throughout development and adulthood (using *GMR-Gal4* promoter): human Tau 0N4R isoform **(B)**, human secreted tandem oligomerising amyloid-β42 **(C)**, or mnb isoform H **(D)**. **(E)** Treatment of flies with PST-001 DYRK1A inhibitor had no effect on eye size of control (*GMR-Gal4/+*) flies **(E)** but suppressed the reduction in eye size of the degenerative mutants that overexpressed in the eye: human Tau **(F)**, tandem amyloid-β42 (tAβ42) **(G)** or mnb **(H)**. **(I)** Degeneration of the eye was quantified by measuring surface area of the eyes [*n* = 7; mean ± Standard error of the mean (SEM)] with flies overexpressing in the eye Tau, Aβ42 or mnb being smaller than control, but there is no difference between mnb and control after treatment with PST-001 (^###^ *p* < 0.001, 2-way ANOVA with Dunnett’s post hoc multiple comparisons test). Furthermore, feeding flies food containing 334 μM of the DYRK1A inhibitor PST-001 increased the eye size of all the degenerative mutants (**p < 0.01, ***p < 0.001, 2-way ANOVA with Bonferroni’s multiple comparisons test).

Overexpression of human DYRK1A, amyloid-β and Tau 0N4R occurs throughout development and adulthood leading to AD-DS pathology and dementia in young adults (Wiseman et al., 2015; Zigman, 2013; Anderson-Mooney et al., 2016; O'Leary et al., 2018). To confirm and compare the neurotoxic effects of these genes in *Drosophila* (Kim et al., 2016; Higham et al., 2019a; Lowe et al., 2019), we used targeted overexpression of a human secreted oligomerising human amyloid-β42 (Aβ42) neuropeptide, human Tau 0N4R or the fly orthologue of DYRK1A called *minibrain* [*mnb* isoform h, which is the neuronal full length isoform robustly overexpressed in neurons (Hong et al., 2012; Gramates et al., 2017; Zerbino et al., 20182018)] in the eye throughout development and adulthood using the *Glass multimer reporter* (*GMR-GAL4*) driver (Higham et al., 2019a; Lowe et al., 2019). Compared to the large semi-crystalline structure of the wild type control (*GMR-GAL4/+,* Figure 2A), overexpression of the neurotoxic genes: Tau (*GMR>Tau,* Figure 2B), Aβ42 (*GMR>Aβ42,* Figure 2C) and *mnb* (*GMR>mnb,* Figure 2D) caused the semi-random loss of photoreceptors neurons resulting in the misalignment of the regular array of these neurons causing a disorganised compound eye or “rough eye” phenotype. The loss of cells in flies overexpressing in the eye human Aβ42, human Tau or mnb could be quantified as a significant reduction in eye surface area compared to control (Figure 2I). To see if mnb inhibition suppressed this degenerative phenotype, flies were fed 334 μM PST-001 throughout their development and adulthood. This was found not to affect the size or integrity of control eyes (Figure 2E). However, PST-001 did increase the eye size of all the degenerative mutants, including partially pharmacologically rescuing the decreased eye size of those overexpressing human Tau (Figure 2F) and human Aβ42 (Figure 2G) to a significantly larger size eye but which remained smaller than the control. Interestingly, the DYRK1A inhibitor was able to fully rescue the reduction of eye size of flies overexpressing of the fly ortholog of DYRK1A, mnb to a level indistinguishable from wild type (Figure 2H), consistent with PST-001 inhibiting mnb.

### The PST-001 DYRK1A Inhibitor Extends the Shortened Lifespan of Alzheimer Disease-Down Syndrome Model Flies

**FIGURE 3:**
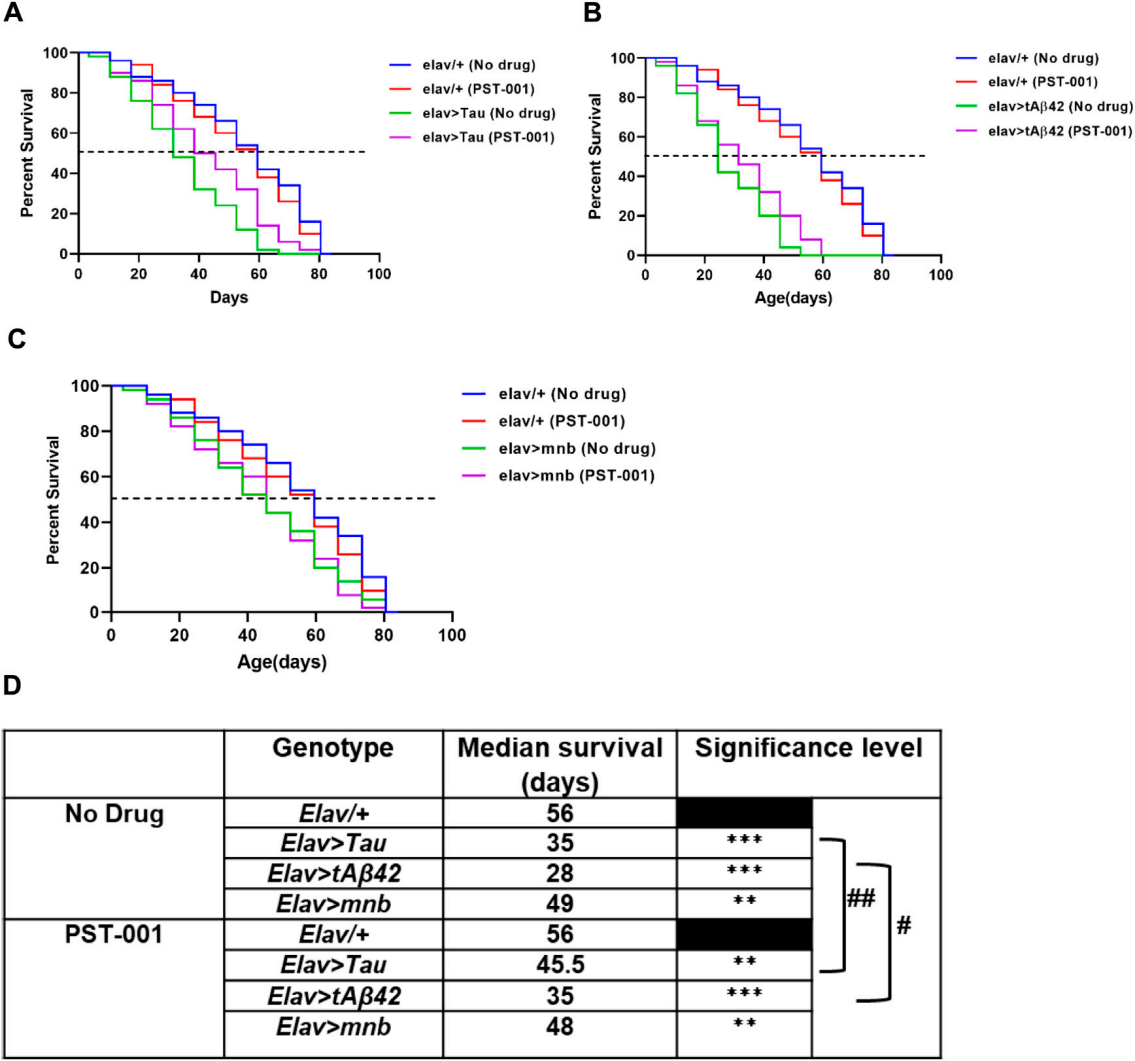
PST-001 DYRK1A inhibitor pharmacologically rescues the reduction of lifespan of *Drosophila* with pan-neuronal expression of human Alzheimer disease-Down syndrome genes. Mantel-Cox (Log-rank) survival plots show effects of overexpression of pan-neuronally expressed (*elav-GAL4*): **(A)** Tau, **(B)** tAβ42 or **(C)** mnb compared to control (*elav-GAL4/+*) fly’s lifespan on food containing no drug or 334 μM PST-001. **(D)** The table shows significant reductions in the lifespan and the median survival (days) with pan-neuronal overexpression of Tau, tAβ42 or mnb (**p < 0.01, ***p < 0.001) compared to control. PST-001 extended the lifespan of human Tau and tAβ42 expressing flies (^#^ *p* < 0.05, ^##^ *p* < 0.01). *n* = 50 flies for all genotypes.

We next wished to test if the reduction of neurotoxicity conferred by the DYRK1A inhibitor could have further beneficial effects on our fly models of AD-DS, which is associated with a significant reduction in lifespan (Querfurth and LaFerl a, 2010; Selkoe and Hardy, 2016; Mukhopadhyay and Banerjee, 2021). Therefore, we overexpressed human Aβ42, human Tau or mnb in all neurons throughout development and adulthood using the *elav-Gal4* promoter to confirm and compare their neurotoxic effect of shortening lifespan (Higham et al., 2019a; Lowe et al., 2019). Compared to wild type flies (*elav/+*) which lived 56 days after hatching (Figure 3D), flies pan-neuronally overexpressing human Tau (38% shorter than wildtype lifespan; Figures 3A,D), Aβ42 (50% shorter; Figures 3B,D) or mnb (13% shorter; Figures 3C,D) had shortened lifespans. Treatment with PST-001 did not affect the lifespan of control (Figures 3A–D) or mnb overexpressing flies (Figure 3C) but did cause a significant extension of lifespan in flies overexpressing human Tau which live 30% longer (Figures 3A,D) or Aβ42 that lived 25% longer (Figures 3B,D) respectively.

### PST-001 DYRK1A Inhibition Improves Motor Deficits of Alzheimer’s Disease-Down Syndrome Model Flies

**FIGURE 4:**
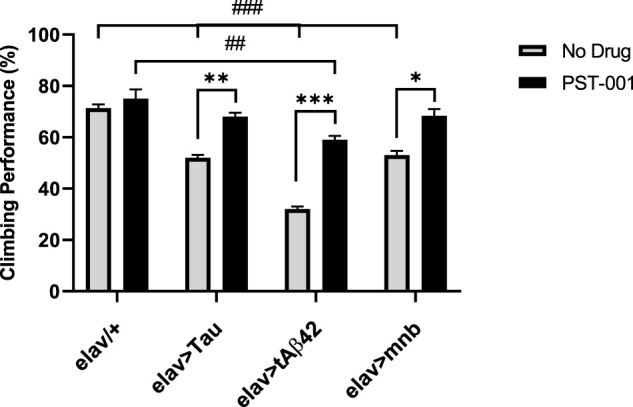
Pharmacological inhibition of DYRK1A rescues movement deficits of the Alzheimer disease-Down’s syndrome model flies. Without drug, pan-neuronal overexpression of Tau, tAβ42 or mnb decreased locomotion compared to control, however after treatment with 334 μM of the DYRK1A inhibitor PST-001 there became no difference between flies overexpressing Tau or mnb compared to control (^##^ *p* < 0.01, ^###^ *p* < 0.001, 2-way ANOVA with Dunnett’s post hoc multiple comparisons test). PST-001 treatment significantly enhanced the climbing performance of all three degenerative mutants (*p < 0.05, **p < 0.01, and ***p < 0.001. 2-way ANOVA with Bonferroni’s multiple comparisons test). Therefore, 334 μM PST-001 fully rescued pan-neuronal Tau and mnb motor deficits and partially rescued tAβ42 deficits. Bars show average performance of 50 flies per genotype.

In order to compare the effects of the different behavioural effects of the neurotoxic genes and test if the DYRK1A antagonist treatment could improve behavioural deficits associated with AD-DS (Querfurth and LaFerl a, 2010; Wiseman et al., 2015; Ballard et al., 2016), we measured a locomotor response assessed using the negative geotaxis assay in young (2–5 days post hatching) flies which was shown to be decreased by overexpression of these genes (Higham et al., 2019a; Lowe et al., 2019). When wild type flies are tapped to the bottom of a 10 cm tube, a negative geotaxis reflex is initiated which causes the flies to move away from gravity up the side of the tube, about 75% of flies were able to climb past a line drawn 2 cm from the top of the tube within 10 s (Figure 4). In contrast, flies with pan-neuronal expression of any of the neurotoxic genes caused a significant reduction (*p* < 0.001, 2-way ANOVA with Dunnett’s post hoc multiple comparisons test) in this locomotor response compared to control. Treatment of the flies with the DYRK1A inhibitor fully rescued locomotor performance of human Tau and mnb flies to a level indistinguishable from control. The DYRK1A inhibitor only partially rescued flies overexpressing human Aβ42, with their performance being significantly greater than when untreated but remaining less than the wildtype control (Figure 4).

### PST-001 DYRK1A Inhibition Improves the Loss of Sleep That Occurs in Alzheimer Disease-Down Syndrome Model Flies

**FIGURE 5:**
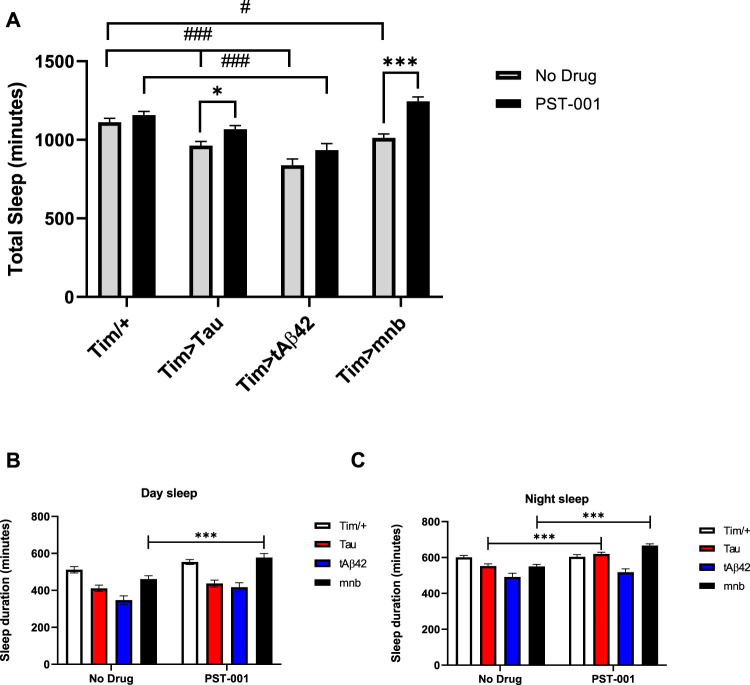
Effect of DYRK1A inhibitor PST-001 on sleep of flies with clock-wide overexpression of tAβ42, Tau or *mnb*. **(A)** Overexpression of Tau, tAβ42 or mnb throughout the clock using *timeless (tim)-GAL4/+* caused a reduction in total sleep compared to control (*tim/+*), whereas only flies expressing tAβ42 displayed a significant loss of total sleep after 334 μM PST-001 treatment (^#^ *p* < 0.05, ^###^ *p* < 0.001, 2-way ANOVA with Dunnett’s post hoc multiple comparisons test). Therefore, PST-001 treatment caused a significant increase in total sleep of flies with clock overexpression of Tau or mnb (*p < 0.05, ***p < 0.001, 2-way ANOVA with Bonferroni’s multiple comparisons test) to control level. The total amount of sleep was measured in the day **(B)** and night **(C)**, PST-001 treatment was found to increase both the loss of day and night sleep of mnb flies compared to just increasing the amount of nocturnal sleep of Tau flies (*p < 0.05 and ***p < 0.001. 2-way ANOVA with Bonferroni’s multiple comparisons test).

Disrupted sleep is both a symptom of AD and AD-DS as well as being known to accelerate disease pathology (Wiseman et al., 2015; Ballard et al., 2016; Musiek and Holtzman, 2016; Holth et al., 2019). Therefore, we wish to compare the effect of our different fly disease models on sleep using *Drosophila* activity monitoring (DAM) which measured activity *via* counting the number of beam-crosses each fly makes under different lighting regimes. Sleep was defined by greater than 5 min inactivity within a 30-min period with males mostly sleeping at night but also taking a “siesta” during the afternoon. Flies with clock wide expression of Aβ42 have previously been shown to decrease circadian rhythms (Chen et al., 2014a). While flies with clock wide expression of Tau increased clock neuron excitability, decreased circadian rhythms, and caused loss of day and night sleep (Arnes et al., 2019; Buhl et al., 2019). Flies overexpressing human Tau, Aβ42 or mnb throughout the clock using the *Timeless (tim)-GAL4* promoter showed a reduction in total (Figure 5A), day (Figure 5B) and night (Figure 5C) sleep. We then tested if the PST-001 DYRK1A inhibitor could rectify these AD model phenotypes and showed DYRK1A inhibition was able to increase total sleep of human Tau or *mnb* clock overexpressing flies (Figure 5A) to wild type levels, thereby demonstrating full pharmacological rescue. When splitting total sleep into day (Figure 5B) and night (Figure 5C) sleep, it became apparent that PST-001 was rescuing the nocturnal loss of sleep of human Tau overexpressing flies, while it was able to increase sleep throughout day and night of the mnb overexpressing flies. PST-001 was not able to increase sleep in human Aβ42 overexpressing flies.

### PST-001 DYRK1a Inhibition Rescues Memory Loss of Alzheimer Disease-Down Syndrome Model Flies

**FIGURE 6:**
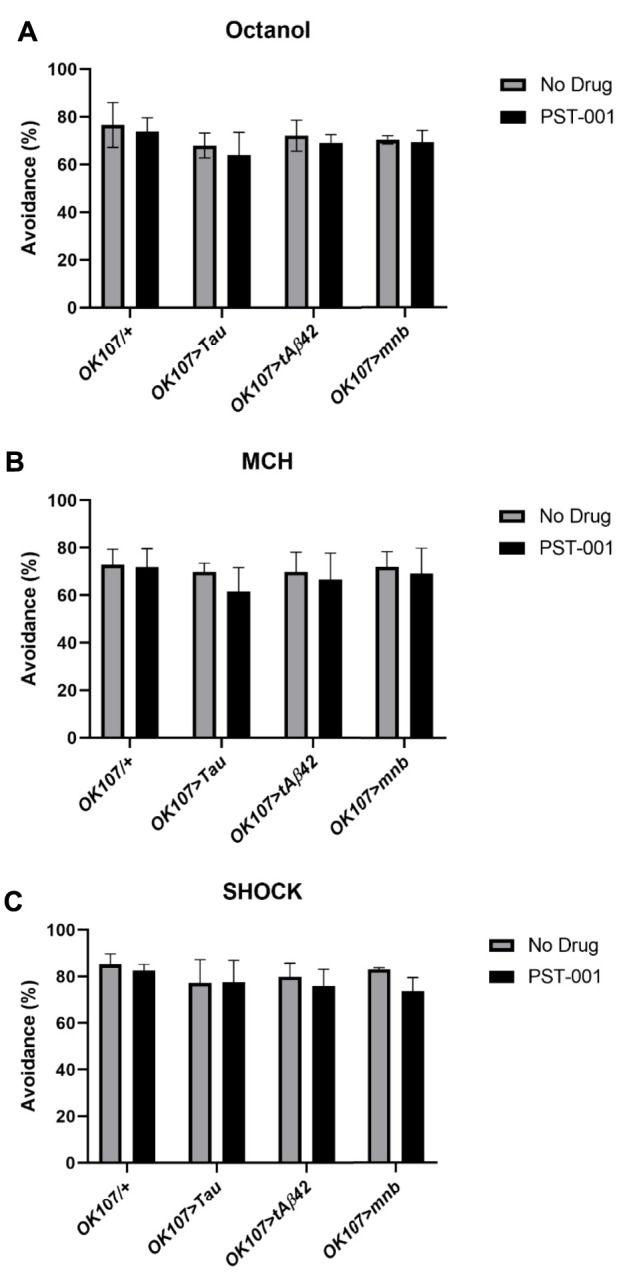
DYRK1A inhibitor PST-001 does not affect sensory behaviour of Alzheimer’s disease-Down’s syndrome models. Neither MB (*OK107-GAL4*) overexpression of Tau, tAβ42 or mnb or PST-001 treatment changed the response of the fly to **(A)** octanol, **(B)** MCH or **(C)** shock compared to control (*OK107/+*) (2-way ANOVA with Dunnett’s post hoc multiple comparisons test). Average % avoidance was taken from 3-4 independent experiments with each *n* = 30–50 flies per experiment per genotype.

Another hallmark of DS and AD is learning and memory difficulties (Querfurth and LaFerl a, 2010; Ballard et al., 2016). Therefore, to compare the effects of the different neurotoxic genes we measured 1 h memory using the olfactory shock assay, with mnb, Tau, and Aβ42 having previously been shown to reduce learning and memory in flies (Tejedor et al., 1995; Iijima et al., 2004; Papanikolopoulou and Skoulakis, 2015; Higham et al., 2019a; Higham et al., 2019b). In this task the flies were exposed to two consecutive odours with the first odour being delivered at the same time as a mild foot shock, and the second odour without shock. After an hour, the flies are taken to a choice point of a T-maze with one arm containing the odour previously paired with shock and the other the non-shocked odour, the flies show learning and memory by avoiding the odour previously paired with shock (Higham et al., 2019a; Higham et al., 2019b). For the flies participate in the assay they need to be able to smell and react to shock normally. Therefore we performed sensory control experiments (Higham et al., 2019a; Higham et al., 2019b) that showed all genotypes either on or off drugs could detect and react to the different odours (octanol, Figure 6A, 4-methylcyclohexanol (MCH), Figure 6B) and shock (Figure 6C) in a manner indistinguishable from untreated control.

**FIGURE 7:**
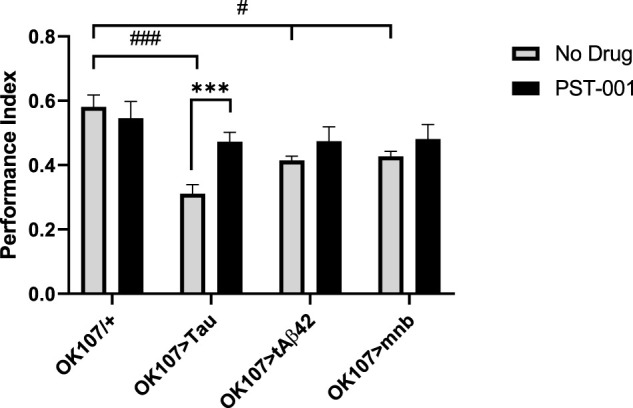
DYRK1A inhibitor PST-001 rescues loss of memory of Alzheimer disease-Down’s sydrome models. Mushroom body (*OK107-GAL4*) overexpression of mnb, tAβ42 or Tau caused a significant decrease in memory (PI) compared to control (*OK107/+*) (^#^ *p* < 0.05, ^###^ *p* < 0.001, 2-way ANOVA with Dunnett’s post hoc multiple comparisons test). 334 μM PST-001 was found to increase Tau mediated memory performance and return all mutant genotypes memory performance to levels indistinguishable from control. (***p < 0.001. 2-way ANOVA with Bonferroni’s multiple comparisons test). Average memory performance index (PI) was taken from four independent experiments with each *n* = 30–50 flies per experiment per genotype.

We found overexpression of human Tau, Aβ42 or mnb throughout the fly memory centre the mushroom body (MB) using the *OK107-GAL4* promoter reduced (*p* < 0.05, 2-way ANOVA with Dunnett’s post hoc multiple comparisons test) 1 h memory compared to control (Figure 7). Treating the flies with the PST-001 DYRK1A inhibitor improved the memory performance of all AD model flies to levels indistinguishable from control. Therefore, the AD model flies are bona fide memory mutants as opposed to flies that cannot detect or respond to the cues and PST-001 is able to enhance their cognition without interfering with normal sensory and reinforcement processing.

## Discussion

We compared the effect of neuronal overexpression of the fly ortholog of DYRK1A called mnb, human Tau or human Aβ42 and found they caused a range of AD and AD-DS relevant phenotypes in *Drosophila* including degeneration of the photoreceptor neurons, shortened lifespan, motor impairment, sleep, and memory loss. The human AD causal genes were more phenotypically extreme than fly mnb overexpression. We found treatment with the DYRK1A inhibitor PST-001 (Stensen et al., 2021a), caused an *in vivo* reduction of phosphorylation of panneuronally expressed human Tau at S262, a site previously identified as being phosphorylated by DYRK1A leading to AD pathology (Azorsa et al., 2010; Frost et al., 2011; Tenreiro et al., 2014). Therefore PST-001 is an inhibitor of the kinase activity of DYRK1A and its fly ortholog, mnb. PST-001 was effective at suppressing the disease relevant phenotypes across models without any detectable adverse effect on control flies. Consistent with PST-001 leaving enough endogenous mnb activity for wildtype functions in the assays studied but still being effective at inhibiting the high levels of kinase activity in the mnb overexpressing flies thereby rescuing its mutant phenotypes. Likewise, PST-001 was effective at ameliorating the disease-relevant phenotypes of human Tau overexpressing flies due to the reduction in pathological phosphorylation of S262 by mnb. Likewise, the abililty of PST-001 to rescue the same mutant phenotypes caused by human Aβ42 overexpression is likely due to kinase inhibition of mnb rescuing the pathological phosphorylation, processing and neurotoxic effects of the Aβ42 peptide, as occurs in other systems (Ferrer et al., 2005; García-Cerro et al., 2017; Arbones et al., 2019). The Western and phenotypic suppression data is consistent with 334 μM PST-001 not being able to completely inhibit mnb hence leaving enough endogenous mnb activity for wildtype functions in the assays studied hence the lack of deficits in the treated controls. Previous work suggests that PST-001 shows inhibition across the DYRK-family (human DYRK1A, DYRK2 and DYRK3) as well as being effective associative memory deficits in Ts65 Dn DS model mice (Stensen et al., 2021b), this and our data therefore is consistent PST-001 being highly effective against *Drosophila* DYRK1A (the product of the *mnb* gene).

This study provides important information on not only the function of mnb, but also the relative pathological consequences of overexpression of AD-associated human Aβ42 and Tau and the potential of DYRK1A inhibitor to treat these deficits. We found that overexpression of AD-associated human Tau 0N4R caused the greatest degeneration of the photoreceptors causing a reduction of ∼60% in eye size compared to control, with Aβ42 also being neurotoxic decreasing the eye by ∼50%, while overexpression of fly mnb caused more modest cell loss reducing the eye size by ∼25%. This confirms previous studies suggesting a link between misexpression of *DYRK1A* with eye size and neurodegeneration (Wegiel et al., 2008; Wegiel et al., 2011; Laguna et al., 2013; Duchon and Herault, 2016; García-Cerro et al., 2017; Watson-Scales et al., 2018), which then drives the accelerated decline in motor and cognitive function in DS and AD-DS and shortened lifespan all strongly correlated with such histopathological changes in the brain (Buchman and Bennett, 2011; Choong et al., 2015; Wiseman et al., 2015; Anderson-Mooney et al., 2016; Arbones et al., 2019). We found that treating flies overexpressing Tau, Aβ42 or mnb in the eye with a DYRK1A inhibitor suppressed degeneration such that their eyes were ∼20%–30% bigger than the equivalent untreated genotype. Therefore, not only did the DYRK1A inhibitor suppress neurodegeneration caused by the overexpression of mnb, but it was also able to suppress amyloid and Tau pathology *via* decreasing endogenous mnb phosphorylation, reducing the neurotoxicity of these genes. This suggests that the PST-001 DYRK1A has therapeutic potential for AD-DS.

We found DYRK1A inhibition was also able to suppress the shortened life associated with pan-neuronal overexpression of AD-associated Tau or Aβ42, again this was only a partial rescue with the treated flies living longer than their equivalent untreated genotype control but not as long as completely normal flies. Intriguingly this lifespan extension was not present in flies overexpressing mnb treated with the DYRK1A inhibitor PST-001, suggesting that the DYRK1A inhibitor may be able to directly suppress human Tau and Aβ42 pathological effects on senescence, further work will be required to elucidate these mechanisms.

These neurotoxic degenerative effects caused by overexpression of Tau, Aβ42 or mnb also had behavioural consequences when expressed in neurons. Firstly, the ability for flies to perform a co-ordinated negative geotaxis climbing response was greatly reduced by the disease associated genes, with DYRK1A inhibition being able to improve the performance of all genotypes. The drug was shown to completely behaviourally rescued the Tau and mnb overexpressing flies, such that their performance was indistinguishable from wildtype flies. Again, we do not know why DYRK1A inhibition showed differential benefits on the different models, but it is possible that this may be due to the transgenes being expressed in different types of neurons e.g., eyes (*GMR-GAL4*) compared to all neurons (*elav-GAL4*), suggesting flies may display selective vulnerability to the neurotoxic effects of the different transgenes. It should also be noted that pan-neuronal Aβ42 overexpression caused the most extreme (∼60%–70%) reduction in motor performance, and DYRK1A inhibition caused the greatest benefit (∼40% improvement), therefore there may be a potential floor effect, whereby the magnitude of the deficit caused by Aβ42 was too great for the drug treatment to completely return the genotype to wildtype.

We also found a similar differential sensitivity to DYRK1A treatment between the genotypes for treatment of the sleep loss caused by clock wide overexpression of the disease-associated genes. Clock expression of Tau, Aβ42 or mnb all caused sleep loss both in the day and night with PST-001 treatment fully rescuing sleep loss of mnb or Tau overexpression. Interestingly when the total sleep was split into day and night sleep, PST-001 was found to suppress day and night sleep loss of mnb flies, as opposed to treating just the nocturnal sleep loss of tauopathic flies. Strikingly people with AD also have similar difficulty sleeping at night (Aldrich et al., 1989; Vitiello and Borson, 2001) making them susceptible to nocturnal wandering (Logsdon et al., 1998). Our work also complements and extends previous work on the effect of clock expression of Tau and Aβ42 on circadian rhythms and sleep disruption in flies (Chen et al., 2014a; Blake et al., 2015; Cassar and Kretzschmar, 2016; Gerstner et al., 2016; Kim et al., 2018; Arnes et al., 2019; Buhl et al., 2019; Cassar et al., 2020). For instance, flies pan-neuronally expressing the Arctic mutant of Aβ42 sleep less during the day and night (Tabuchi et al., 2015), while expression of tandem Aβ42 caused behavioural arrhythmia (Chen et al., 2014a).

Our work also demonstrates the potential of DYRK1A inhibitors as sleep correctors for DS and AD-DS which may slow pathology and boost cognition, the high throughput capability of flies will allow further characterisation and screening of DS and AD circadian and sleep drugs (Dissel et al., 2017; Wang et al., 2020). People with DS also display disrupted sleep including sleep apnoea which can negatively impact their cognition and motor control (Chawla et al., 2020). Likewise, many people with AD in addition to the disrupted sleep at night mentioned previously also display sun-downing, where upon the patient experiences more anxiety and confusion in the evening (Vitiello et al., 1992; McCurry et al., 1999; Volicer et al., 2001). Insomnia, nocturnal wandering and not remembering where one is, are the primary reasons for eventual institutionalisation of people with AD, resulting in loss of independence, support networks and increased healthcare costs (Pollak and Perlick, 1991).

Furthermore, a robust circadian clock and sleep schedule improves memory function and is required for consolidation of long term memory (Gerstner and Yin, 2010) with post-mortem AD brain slices revealing neurodegeneration of the suprachiasmatic nucleus (SCN) of the hypothalamus which is the location of the mammalian circadian clock. Transgenic mice models of AD also show SCN degeneration (Sterniczuk et al., 2010; Stevanovic et al., 2017). Therefore, flies and mice expressing human 0N4R Tau exhibit behavioural dysfunction and neurophysiological changes, including elevated neuronal activity, which precedes neurodegeneration (Wittmann et al., 2001; Sterniczuk et al., 2010). Like flies (Buhl et al., 2019; Cassar et al., 2020), tauopathic mice show disrupted circadian rhythms and sleep showing neuronal hyperexcitability and inability to sleep at night (Stevanovic et al., 2017). The sleep-wake cycle is known to regulate brain interstitial fluid Aβ42 and tau in mice and cerebrospinal fluid Aβ42 and Tau in humans. Levels increase during the day and are removed at night with sleep deprivation further increasing pathological Tau seeding and spreading (Musiek and Holtzman, 2016; Holth et al., 2019). This reiterates the importance of healthy circadian rhythms and sleep for cognition and improving DS and AD symptoms as well slowing or preventing neurodegeneration in AD and AD-DS.

We also saw cognitive deficits, with MB memory neuron overexpression of Tau, Aβ42 or mnb reducing associative memory performance with PST-001 treatment increasing the memory of Tau overexpressing flies and improving the performance of the other mutant genotypes. This validates the use of PST-001 as a cognitive enhancer in preclinical animal models of DS, AD-DS, and AD. This suggests that DYKR1A inhibitors may be beneficial to people with DS prior to displaying memory impairment associated with AD-DS, allowing targeted treatment prior to the onset of irreversible neurodegeneration, thereby correcting the causes as opposed to just the symptoms of AD.

The results of our study are also consistent with finding that mice overexpressing human *DYRK1A or* mouse *Dyrk1a*, have similar motor and cognitive deficits reinforcing triplication of *DYRK1A* is likely to contribute to these behavioural deficits in DS and AD-DS (Altafaj et al., 2001; Martínez de Lagrán et al., 2004; Ortiz-Abalia et al., 2008; Arque et al., 2013; Souchet et al., 2014; García-Cerro et al., 2017; Watson-Scales et al., 2018). Reiterating that mnb is both molecularly and functionally conserved with DYRK1A. Likewise, this work also reinforces that neuronal overexpression of AD-associated Tau (Wittmann et al., 2001; Folwell et al., 2010; Iijima-Ando and Iijima, 2010; Kosmidis et al., 2010; Beharry et al., 2013; Papanikolopoulou and Skoulakis, 2015; Sealey et al., 2017; Higham et al., 2019a; Higham et al., 2019b; Buhl et al., 2019; Lowe et al., 2019) and Aβ42 (Iijima et al., 2004; Chiang et al., 2010; Spere tta et al., 2012; Chen et al., 2014a; Blake et al., 2015; Ping et al., 2015; Tabuchi et al., 2015; Higham et al., 2019a) result in neurodegeneration leading to a range of disease relevant phenotypes in flies.

It would be interesting to understand further how Tau, Aβ42 and mnb interact to influence neuronal function causing the behavioural changes seen. In addition to neurodegeneration, overexpression of these disease associated genes disrupts intrinsic and synaptic plasticity (Higham et al., 2019a; Higham et al., 2019b; Buhl et al., 2019; Lowe et al., 2019), contributing the loss of memory and sleep reported. Together this work also shows that the fly models replicate many of the key features of rodent transgenic Tau, Aβ42 and DYRK1A models including changes in excitability, increased Ca^2+^ signaling, neurodegeneration, and impaired synaptic plasticity, memory, and sleep (Selkoe, 2012; Spillantini and Goedert, 2013; Spires-Jones and Hyman, 2014; Wang and Mattson, 2014; Booth et al., 2016a; Arendt et al., 2016; Booth et al., 2016b; Kay et al., 2016; Selkoe and Hardy, 2016; Biundo et al., 2018; Arbones et al., 2019). This body of work also highlights how well these behaviours and mechanisms are conserved between flies and mammals including humans, allowing the well-established assays, genetics, and rapid ageing of *Drosophila* to study these processes.

## Conclusion

We showed that *Drosophila* which overexpress mnb, human Tau or Aβ42 in different neuronal populations cause a range of AD and AD-DS relevant phenotypes and pathology including degeneration of the photoreceptor neurons, shortened lifespan, motor impairment, sleep, and memory loss. We demonstrate that the PST-001 DYRK1A inhibitor decreases phosphorylation of human Tau at S262 and is able to suppress disease relevant phenotypes caused by overexpression of mnb, human Tau or Aβ42. This allows researchers to exploit the genetic tractability and short generation time of this non-protected species to study the role of these genes in DS and AD processes and then rapidly characterise them as potential targets for new drugs to reverse these disease-relevant deficits.

## Data Availability

Underlying data are openly available from Dryad under the DOI: https://doi.org/10.5061/dryad.z08kprrg8

## Abbreviations

AD, Alzheimer’s disease; AD-DS, Alzheimer’s disease-down syndrome; ANOVA, analysis of variance; APP, amyloid precursor protein; CS^+^, conditioned stimulus; DAM, *Drosophila* activity monitoring; DD, continuous darkness; DS, down syndrome; DYRK1A, dual specificity tyrosine-phosphorylation-regulated kinase 1A; GMR, glass multimer reporter; HSA21, human chromosome 21; ITM, intermediate-term memory; LD, 12 h light:12 h dark conditions; *MAPT* gene, microtubule associated protein tau; MB, mushroom body; MCH, 4-methylcyclohexanol; mnb, minibrain; PI, performance index; SEM, standard error of the mean; tAβ42, tandem oligomerising human Amyloid-β42; tim, timeless; US, unconditioned stimulus.
